# Multimodal Laser Stimulation and Traditional Needle Acupuncture in Post-Stroke Patients—A Pilot Cross-Over Study with Results from Near Infrared Spectroscopy

**DOI:** 10.3390/medicines6040115

**Published:** 2019-12-16

**Authors:** Gerhard Litscher, Xiaoning Zhang, Zemin Sheng, Xiang-Hong Jing, Lu Wang

**Affiliations:** 1 Research Unit of Biomedical Engineering in Anesthesia and Intensive Care Medicine, Research Unit for Complementary and Integrative Laser Medicine, and Traditional Chinese Medicine (TCM) Research Center Graz, Medical University of Graz, 8036 Graz, Austria; zxnruobing@126.com (X.Z.); lu.wang@medunigraz.at (L.W.); 2 Institute of Acupuncture-Moxibustion, China Academy of Chinese Medical Sciences, Beijing 100700, China; jxhtjb@263.net; 3 Department of TCM, Privatclinic Lassnitzhoehe, 8301 Lassnitzhoehe, Austria; shengzemingraz@hotmail.com

**Keywords:** laser stimulation, acupuncture, stroke, near infrared spectroscopy, ear acupuncture, body acupuncture, scalp acupuncture

## Abstract

**Background:** The objective of this pilot study was to evaluate the cerebral effects of laser stimulation and traditional needle acupuncture in patients after stroke. **Methods:** Seventeen stroke patients (12 female and five male; mean age ± SD: 66.5 ± 12.9 years) were randomly selected in a stroke rehabilitation hospital. Patients’ regional cerebral blood oxygen saturation (rSO2) values were recorded before, during, and after needle acupuncture (scalp, ear and body) as well as before, during, and after corresponding laser stimulation (red laser, four points: 100 mW, 658 nm, 500 µm; yellow laser, one point: 50 mW, 589 nm, 500 µm; infrared laser, three points: 100 mW, 810 nm, 500 µm; green laser, one point: 5 mW, 532 nm, 500 µm) in a cross-over study design. **Results:** There were no significant changes after needle acupuncture in the phases immediately after needle insertion or during acupuncture stimulation. However, after manual needle acupuncture and after laser stimulation, the majority of the rSO2 values showed increases. The highest value (+3%) was reached after laser stimulation treatment. Heart rate and blood pressure before and after the treatments did not show significant alterations. **Conclusions:** Changes in local cerebral oxygen saturation could be quantified, although confirmation may only be expected after extensive follow-up studies.

Dear Editors of *Medicines*,

Strokes are life changing events that can both physically and emotionally affect a person. A treatment for recovering lost functions is referred to as stroke rehabilitation. Rehabilitation after a stroke should start as soon as possible and can take from a few days to over a year. Acupuncture has been used for stroke reconstruction for more than two thousand years and is now used in many parts of the world. Laser acupuncture, as a kind of high-tech acupuncture, has recently been attracting more and more attention. However, there are few studies on the mechanisms of acupuncture and laser acupuncture in promoting the recovery of stroke patients. 

Near-infrared spectroscopy (NIRS) uses the near-infrared region of the electromagnetic spectrum (from about 800 to 2500 nm). Medical applications of NIRS include noninvasive measurements of the amount and oxygen content of hemoglobin [1]. The advantage of NIRS is that it can penetrate much deeper into a sample than mid-infrared radiation [2]. 

In the past, we investigated the relationship between acupuncture and cerebral microcirculation by using NIRS. In healthy male volunteers, acupuncture at specific acupuncture points was shown to lead to an increase in oxygenated hemoglobin and in the tissue oxygenation index. However, needling and laser stimulation at non-acupoints was not found to produce the same effect on cerebral oxygenation quantified by NIRS technology [3]. In 16 persons, a significant decrease was found in oxyhemoglobin after needle insertion and stimulation, and this was accompanied by an increase in deoxyhemoglobin [4]. These previous results suggest that NIRS may be useful in visualizing and quantifying the cerebral vascular effects of acupuncture and acupuncture-like stimulation on microcirculation in patients who have had a stroke [5,6]. The aim of this article is to preliminarily evaluate the effects of laser stimulation and traditional acupuncture in post-stroke patients on regional cerebral oxygen saturation in the rehabilitation phase. In the present study, we compare the effects of scalp needle acupuncture, body needle acupuncture, and ear needle acupuncture with corresponding multimodal laser stimulation after a stroke—the first time such a comparison has been done. The aim of such a pilot study is to calculate the effect sizes of these methods in order to design a large clinical study.

Seventeen patients who had experienced a stroke (Chinese diagnosis “Zhong Fang”) were studied in an Austrian private hospital for stroke rehabilitation with a multimodal needle acupuncture scheme, as well as with corresponding multimodal laser stimulation within a three week rehabilitation stay. 

Both treatments lasted 20 min and were administered within one week. The adult patients (12 female and five male) had a mean age ± SD of 66.5 ± 12.9 years (range 43–89 years), a mean height ± SD of 167.8 ± 9.6 cm, and a mean weight ± SD of 74.8 ± 14.3 kg. All patients received needle acupuncture and laser stimulation treatment for post-stroke rehabilitation and were not under the influence of centrally active medication. None of the patients had a body temperature out of the normal range. The study was approved by the local ethics committee in Lassnitzhoehe/Austria, and the noninvasive recording procedure (cerebral oxygen saturation) was performed in accordance to the Declaration of Helsinki. At the latest of one day after cerebral oxygen saturation was monitored, all persons were informed about the nature of the investigation and the results of the own measurement. All patients gave their oral, informed consent for inclusion before they participated in the study. As one limitation, it has to be explicitly mentioned that the preliminary pilot study was not registered as a clinical trial, and the reporting does not fully follow the CONSORT and STRICTA recommendations.

The measurements of the changes in regional cerebral oxygen saturation (rSO_2_) were performed with an INVOS 5100C Oximeter (Somanetics Corp., Troy, MI, USA) instrument. For that reason, near-infrared light (730 and 805 nm) was emitted through the skin of the frontal brain, and, after passing through different kinds of tissue (skin and bone), the reflected light was detected at two distances from the light source (3 and 4 cm). The spectral absorption of the blood in deeper structures (2–4 cm) could be determined and defined as the rSO_2_ values [7,8]. In addition, heart rate (HR) and blood pressure (BP) were recorded before and after the completion of every session to demonstrate stable conditions in both investigations (needle and laser acupuncture).

Within the present cross-over study, a multimodal acupuncture scheme was used. This scheme included body, ear and scalp acupuncture points. Scalp and ear acupuncture was performed on the stroke-affected side; stimulation in the periphery was performed contra laterally. We wanted to have a maximal input of stimulation. Therefore, we chose a new concept that combined body, scalp and ear acupuncture. The different kinds of lasers were chosen according to the different penetration depths (Figure 1).

All participants received needle acupuncture at LI11 (Quchi), LI4 (Hegu), ST36 (Zusanli), GB39 (Xuanzhong), GV20 (Baihui), three points of motor area, and one ear point (Shenmen). Disposable needles (0.30 × 30 mm; Huan Qiu, Suzhou, China) for body and scalp acupuncture and disposable pressing needles (0.22 × 1.5 mm; Wujiang City Cloud & Dragon Medical Device Co., Ltd, Wujiang, China) for ear acupuncture, both single use only, were used for the traditional form of acupuncture. Needles were slowly inserted approximately 0.5–2 cm into every point; equal manipulations of twirling, lifting, and thrusting (in the middle of the treatment session) were performed to reach acupuncture a deqi soreness, heaviness, and distension sensation. 

The same patients received laser stimulation at the same points with different laser parameters. A Weberneedle Combi-Laser system (Weber Medical, Lauenfoerde, Germany) from the Privatclinic Lassnitzhoehe in Austria was used for the laser stimulation. The stimulation at the highest point of the skull (Baihui, GV20) was performed with a yellow laser modality (see Figure 1). The yellow laser was, at that time, only available from Weber Medical and, for research purposes, at the Medical University of Graz and in the Privatclinic Lassnitzhoehe in Austria. As partly shown in Figure 1, the parameters of the laser stimulation were the following: red laser (four acupoints): 100 mW, 658 nm, 500 µm, and penetration depth about 2–4 cm; yellow laser (one acupoint): 50 mW, 589 nm, 500 µm, and penetration depth about 3 cm; infrared laser (three acupoints): 100 mW, 810 nm, 500 µm, and penetration depth about 3–4 cm; green laser (one acupoint): 5 mW, 532 nm, 500 µm, and penetration depth about 0.5–1 cm [9].

Figure 2a shows the procedure and stimulation scheme for both conditions (laser stimulation and needle acupuncture) of the study. Treatment took place from point B to point D. The patients’ rSO_2_ values were recorded at points A–E.

Data were analyzed at the Medical University of Graz with the Sigma Plot 14.0 software (Systat Software Inc., Chicago, IL, USA). The graphical presentation of results uses box plot illustrations. Testing was performed with a one way repeated measures analysis of variance and a Kruskal–Wallis one way analysis of variance on ranks. The criterion for significance was *p* < 0.05.

All patients tolerated needle acupuncture and laser stimulation treatments well, and no adverse effects were observed. The changes of the cerebral rSO_2_ values of all patients during the different conditions are shown in Figure 2b–e.

Both after manual needle acupuncture and after laser stimulation, the majority of the rSO_2_ values showed non-significant increases. The highest value (+3%) was reached after laser stimulation treatment.

HR and BP before and after each session (Pre and Post in Figure 2) showed no significant differences (see Table 1).

As in previous studies [10], control experiments were performed to eliminate the possibility that the evoked potential obtained after laser irradiation was influenced by timed artifacts arising from the interface between the laser instrument and the signal recording system.

The therapy of stroke rehabilitation helps patients to return to normal life as much as possible. Treatment may also include acupuncture, physiotherapy, speech, language therapy, and massages [11].

In our pilot study on 17 patients following a stroke presented here, we used a multimodal needle acupuncture scheme as part of a rehabilitation treatment that simultaneously included scalp acupuncture, ear acupuncture, and body acupuncture. Near infrared spectroscopy is a noninvasive method for measuring rSO_2_ through the intact skull that has been successfully applied in basic medical research and clinical indications for many years [12].

In the literature, it has been reported that scalp acupuncture often produces remarkable results with just a few needles and usually brings about immediate improvement, sometimes taking only several seconds-to-a-minute. That was not the case in our study, at least as far as reflected by the evaluation parameter rSO_2_. There were no significant changes after needle acupuncture in the phases immediately after needle insertion or during acupuncture stimulation. Even after needle acupuncture, the values of regional cerebral oxygen saturation did not significantly increase or decrease. However, it was noticeable that in both hemispheres, on the ipsilateral side as well as on the contralateral side after needling (motoric region) in the frontal near field, increases in blood oxygen metabolism occurred (stimulated side: +2%; non-stimulated side: +1.5%). This trend was similar to the changes after laser stimulation. Here, the largest rSO_2_ increase was on the non-affected side (+3%). In order to demonstrate stable conditions concerning HR and BP, we also recorded these parameters before and after treatment, and we also found no significant differences before and after both kind of treatments. 

If one tries to make a comparison between needle and laser acupuncture in our pilot study, one could say that the needle acupuncture showed more trends towards an increase (non-significant) on the stroke-affected cerebral side than the laser acupuncture. However, the overall trend of an increase of laser treatment was a little bit greater than that of needle stimulation (also non-significant). That interpretation has to be done very cautiously, since, as already mentioned several times, the effects had no significance. 

Here, we are already on a very important topic of our investigations, namely the limitations of the study. The study was designed to be integrated into the clinic’s routine rehabilitation program. This means that we performed the two measurements in each patient that were necessary for a cross-over study in a randomized order on two different days within a week. Additionally, the number of patients *(n* = 17) was not large, resulting in a heterogeneous gender and age distribution. In addition, we cannot quantify whether and, if so, to what extent the activation of the different acupuncture schemes (scalp, ear, and body) contributed to changes in rSO_2_ values. 

It is known that about ten sessions of acupuncture, on average, should be applied to patients for full treatment, but here, in our preliminary investigation, acupuncture was applied only two times (one using needle and one using laser stimulation). In future investigations, which should be performed not during a very limited rehabilitation stay in the hospital, we will apply the acupuncture treatment for at least five-to-seven sessions, enlarge the sample size, minimize patients’ age ranges, and choose only one kind of gender. This maybe may lead to significant results. 

Since intensive research is being carried out regarding the validation of the technical parameters for laser therapy in general and laser acupuncture in particular [12,13], future procedures that are clinically very practical are also to be expected.

Of course, no statement can be made about possible long-term effects, since this would certainly require at least five-to-seven treatment sessions, as mentioned before. This could be the subject of follow-up studies and could potentially be included in a three-week rehabilitation program.

For the first time in a pilot study, a new sophisticated design and a comparison between a multimodal needle acupuncture scheme and corresponding laser acupuncture stimulation in patients after a stroke is presented. Trends in the direction of an increase in local cerebral oxygen saturation levels were detectable; therefore, extensive follow-up studies may be justified.

## Figures and Tables

**Figure 1:**
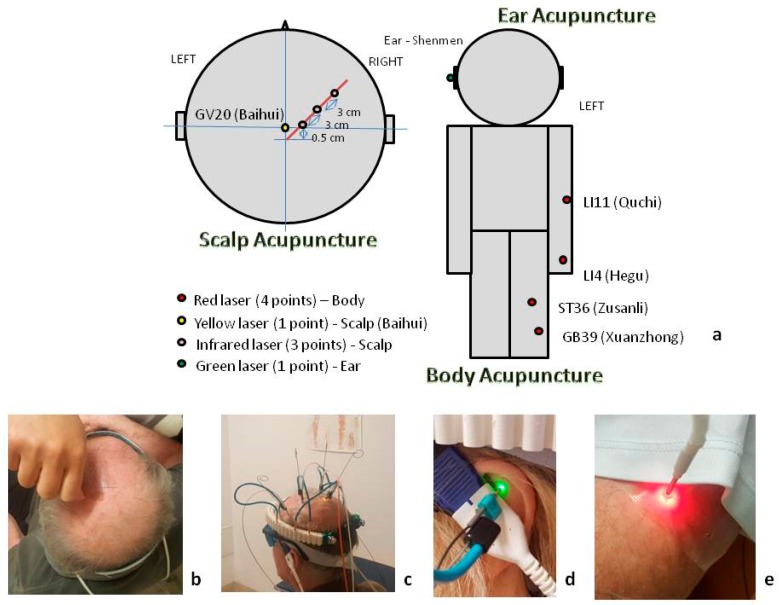
(**a**) Multimodal laser stimulation and needle acupuncture scheme in the present cross-over study. (**b**) Scalp needle acupuncture, (**c**) scalp laser stimulation, (**d**) ear laser acupuncture, and (**e**) body laser stimulation treatment in post-stroke patients.

**Figure 2:**
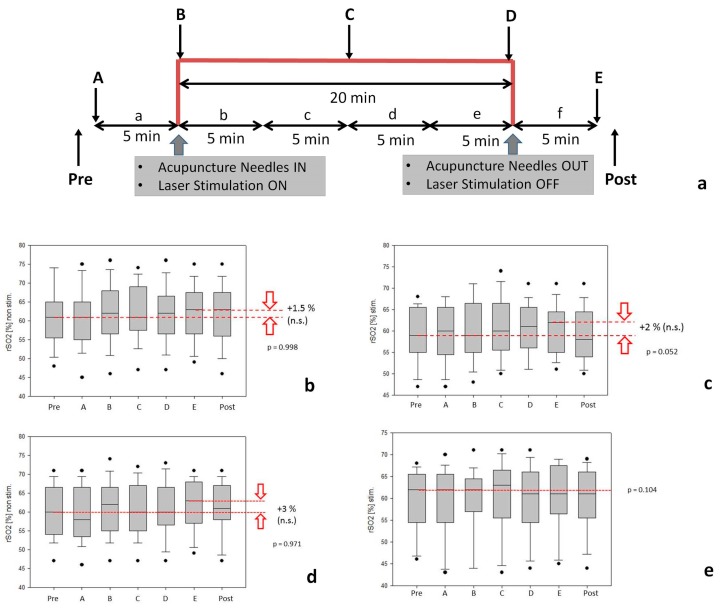
(**a**) Recording procedure for the different stimulation methods (measuring points A–E; measuring phases a–f). (**b**) Box plot diagram of regional cerebral oxygen saturation (rSO_2_) measurement values of all seven different conditions before, during, and after needle acupuncture from the non-stimulated side of the 17 post-stroke patients. The lines in the boxes represent the median, the ends of the boxes define the 25th and 75th percentile, and the error bars define the 10th and 90th percentile. The arrows indicate the increase of the median at the end of the treatment in comparison to the pre-condition interval. (**c**) RSO_2_ measurement values before, during, and after needle acupuncture of the stimulated side. (**d**) RSO_2_ measurement values before, during, and after laser stimulation of the non-stimulated side. (**e**) Cerebral rSO_2_ measurement values before, during, and after laser stimulation of the stimulated side.

**Table 1:** Heart rate (HR), systolic (BPsys) and diastolic (BPdiast) blood pressure before and after needle and laser stimulation. Values are given in mean ± standard deviation (SD).

	Before Needle Stimulation (Pre)	After Needle Stimulation (Post)	Before Laser Stimulation (Pre)	After Laser Stimulation (Post)
HR [1/min]	69.7 ± 10.1	69.7 ± 9.0	72.3 ± 11.4	73.1 ± 10.1
BPsys [mmHg]	144.3 ± 23.9	141.3 ± 16.4	133.4 ± 35.5	136.2 ± 38.3
BPdiast [mmHg]	88.6 ± 8.4	89.7 ± 10.7	89.4 ± 12.6	87.8 ± 13.9
